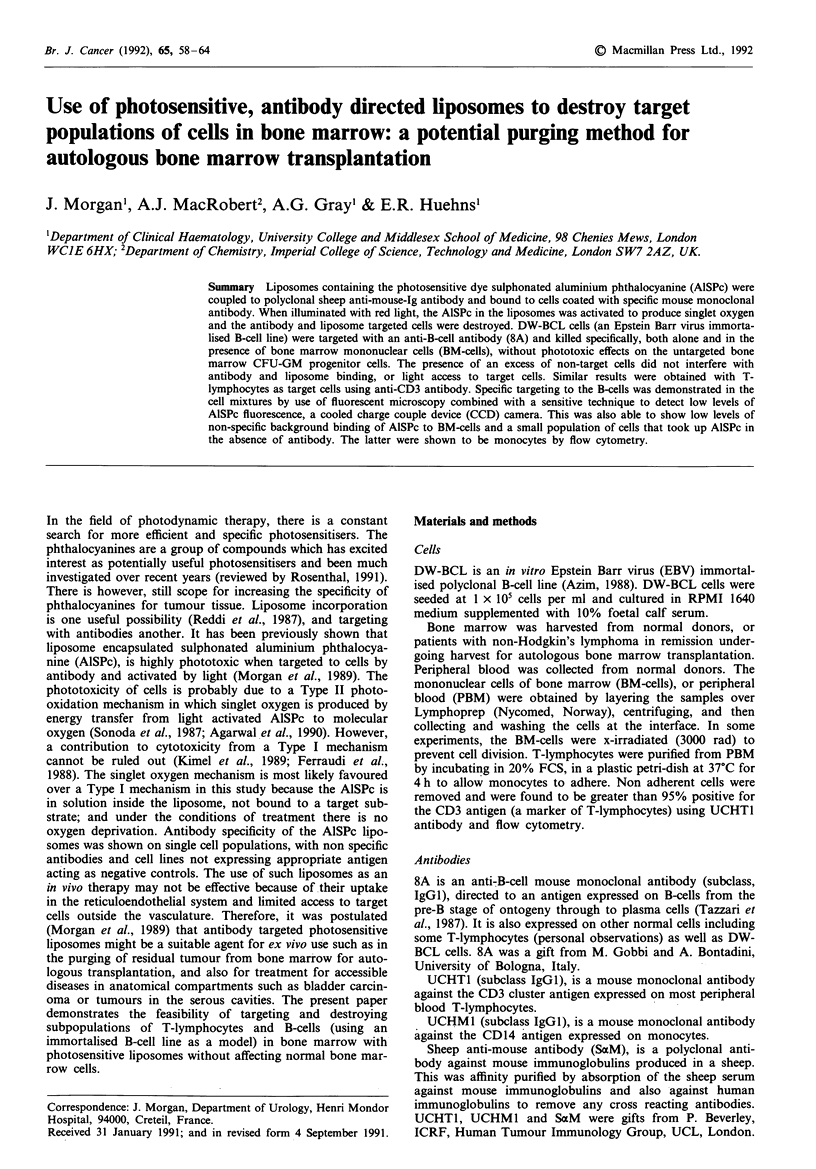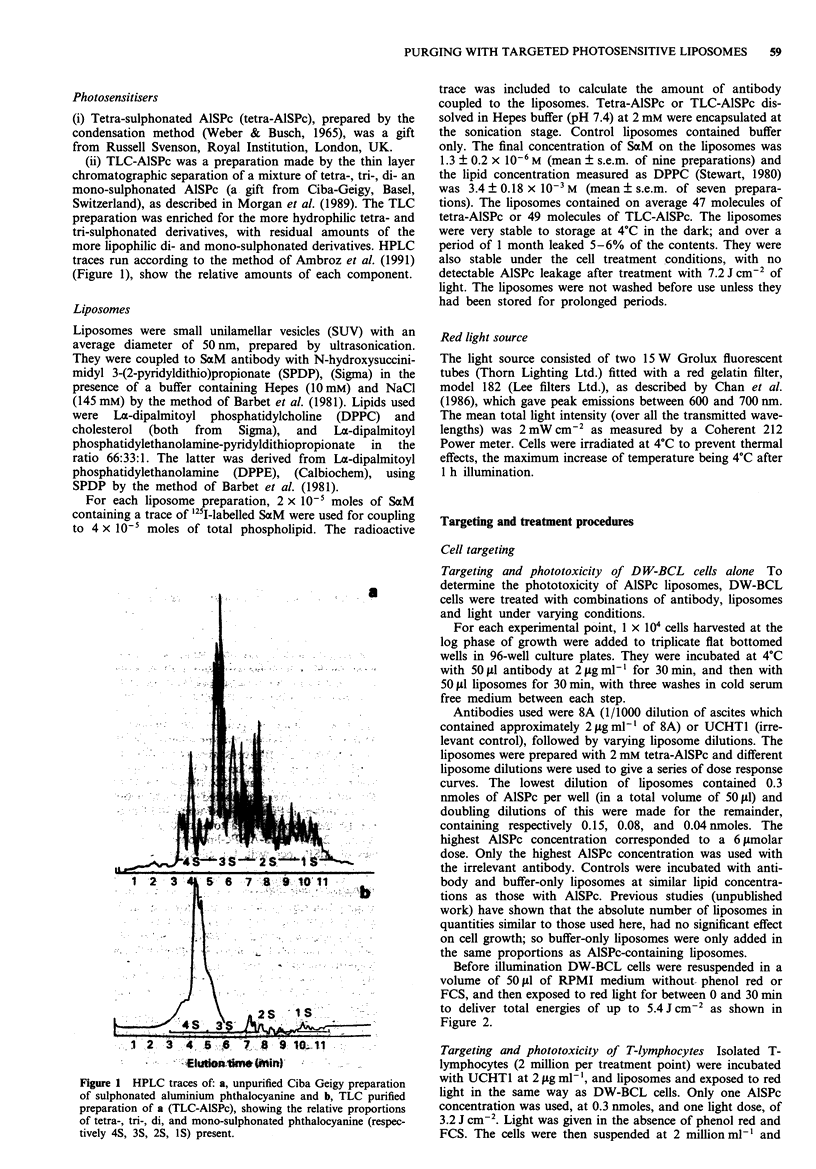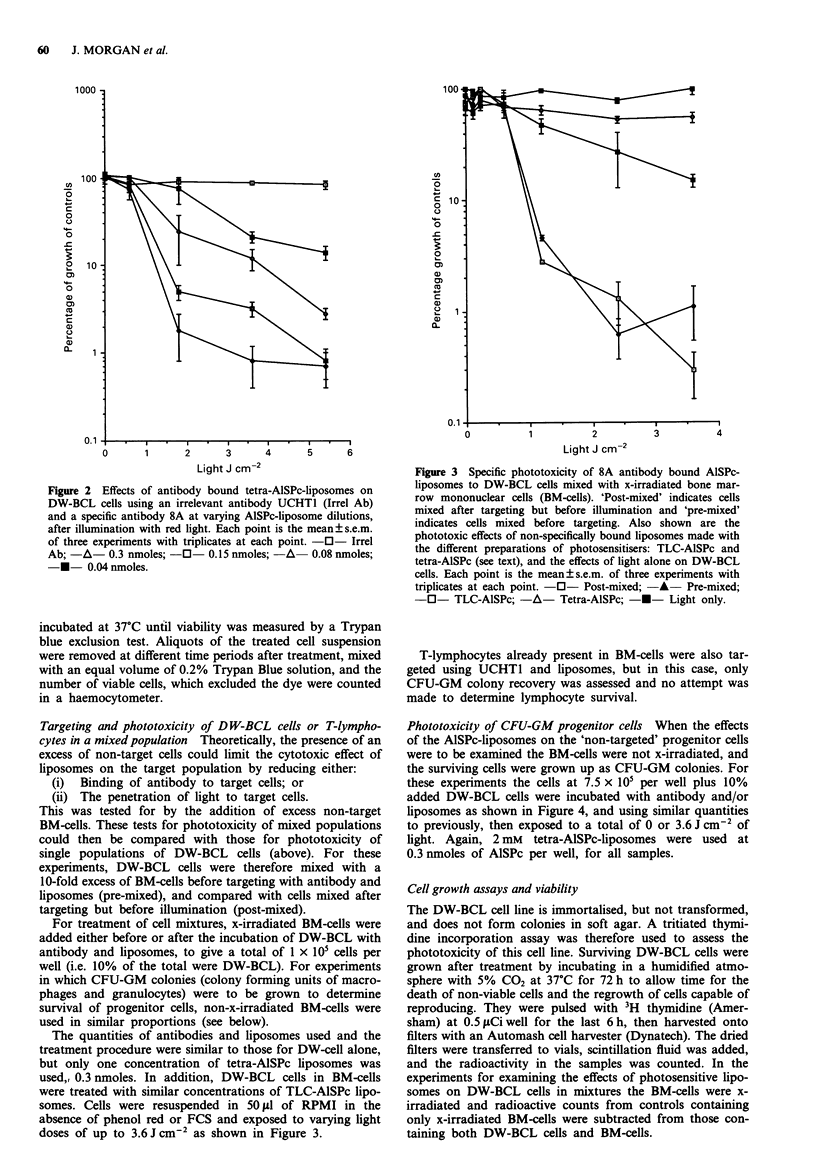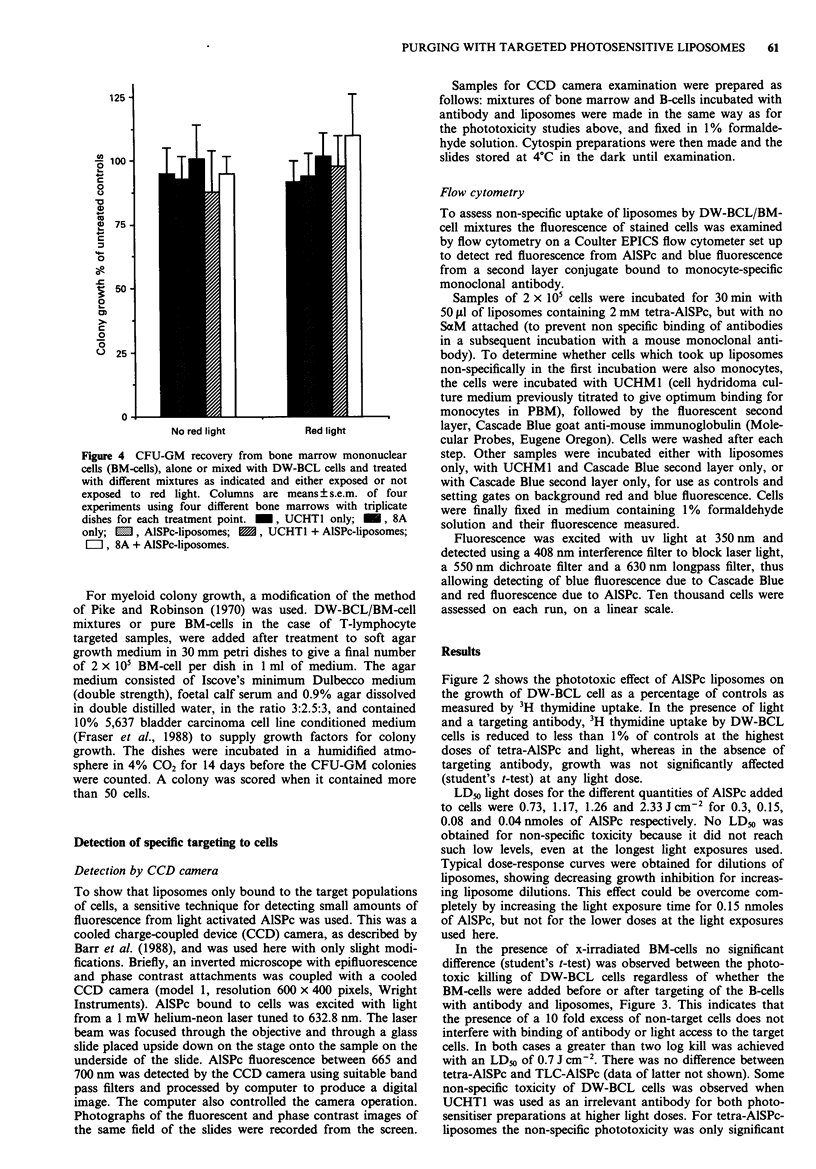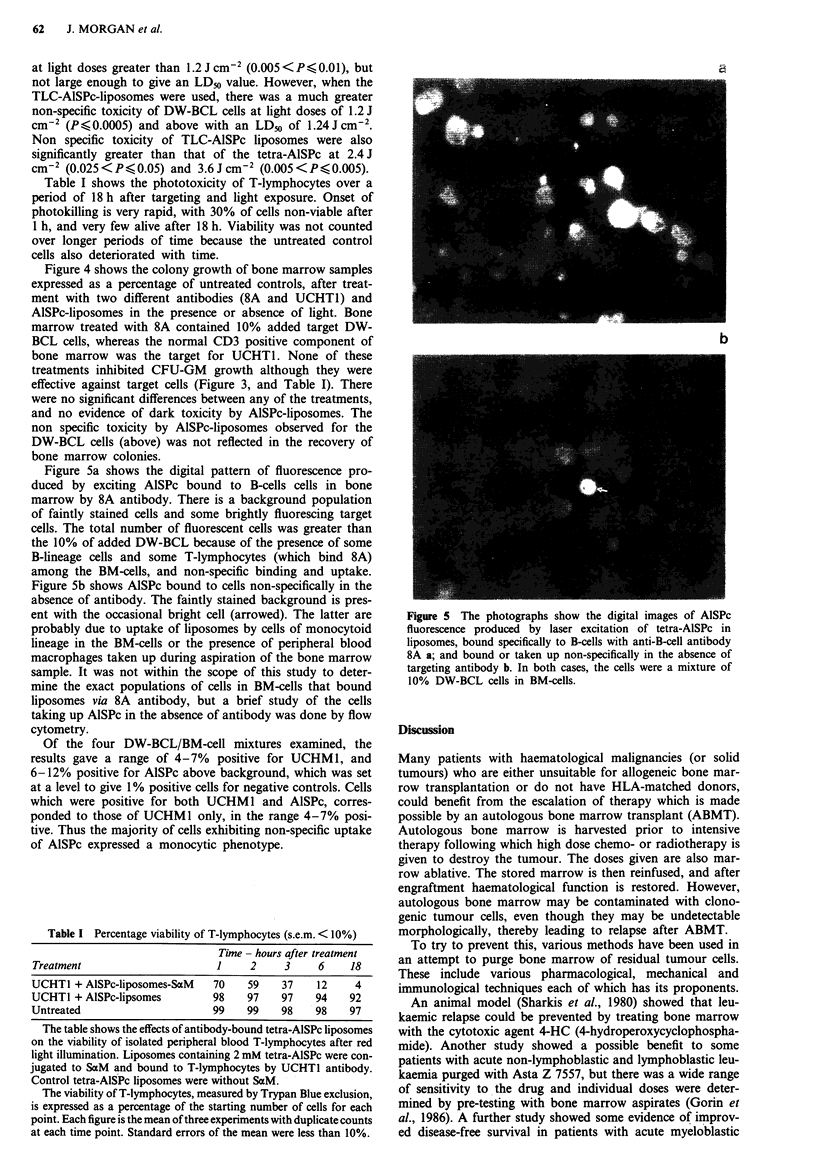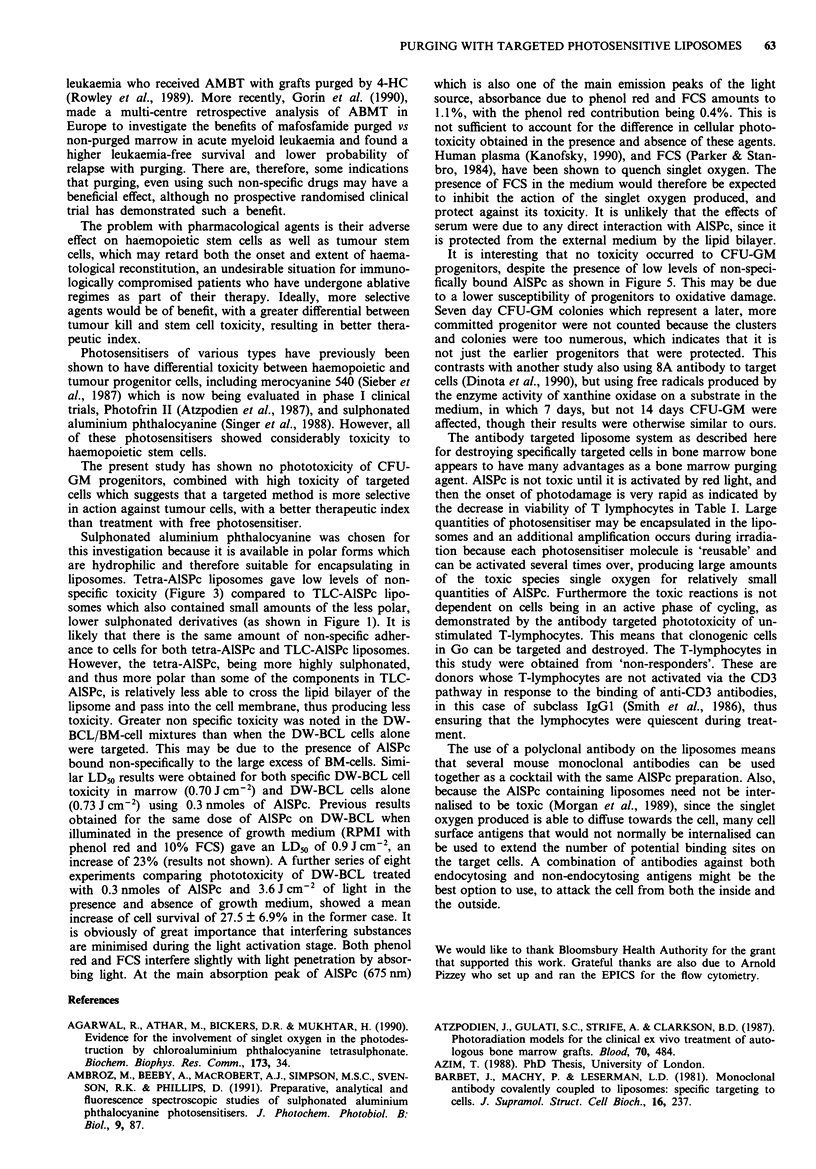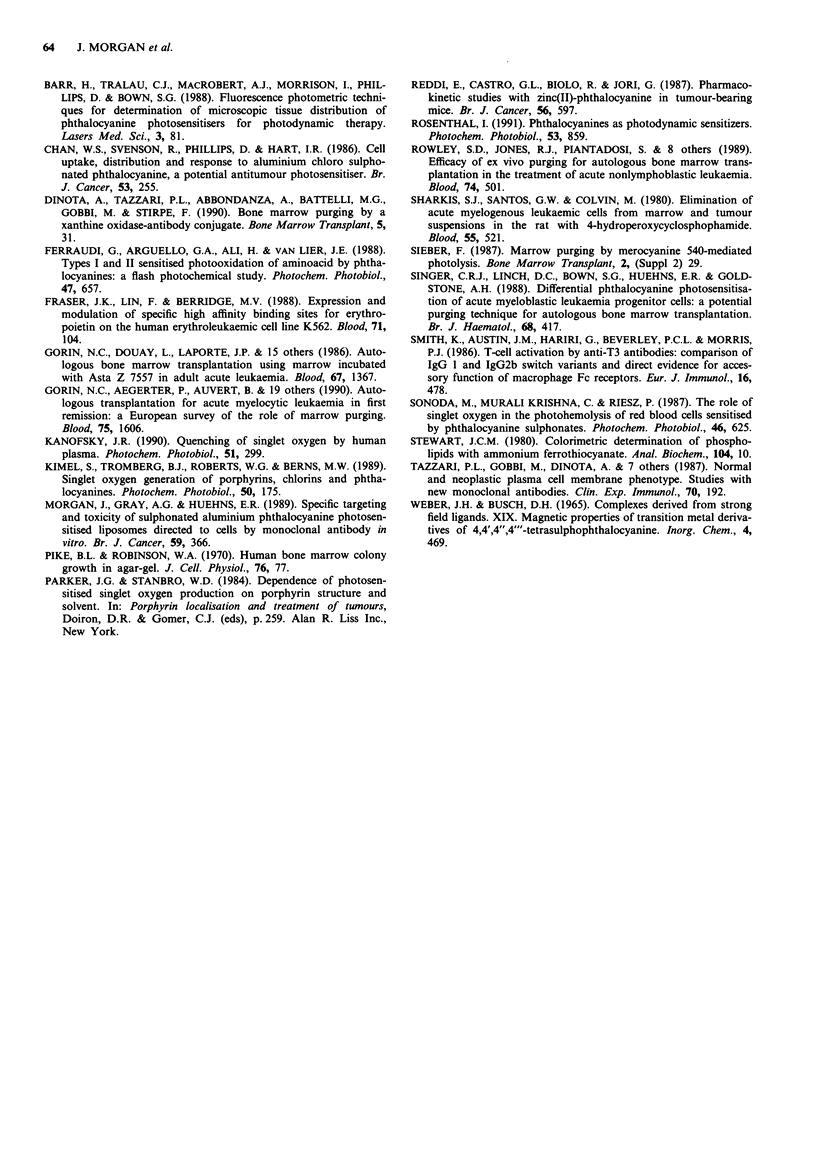# Use of photosensitive, antibody directed liposomes to destroy target populations of cells in bone marrow: a potential purging method for autologous bone marrow transplantation.

**DOI:** 10.1038/bjc.1992.11

**Published:** 1992-01

**Authors:** J. Morgan, A. J. MacRobert, A. G. Gray, E. R. Huehns

**Affiliations:** Department of Clinical Haematology, University College and Middlesex School of Medicine, London, UK.

## Abstract

**Images:**


Br. J. Cancer (1992), 65, 58-64 © Macmillan Press Ltd., 1992

### Use of photosensitive, antibody directed liposomes to destroy target populations of cells in bone marrow: a potential purging method for autologous bone marrow transplantation

J. Morgan[1], A.J. MacRobert[2], A.G. Gray[1] & E.R. Huehns[1]

[1]*Department of Clinical Haematology, University College and Middlesex School of Medicine, 98 Chenies Mews, London WC1E 6HX;* [2]*Department of Chemistry, Imperial College of Science, Technology and Medicine, London SW7 2AZ, UK.*

**Summary** Liposomes containing the photosensitive dye sulphonated aluminium phthalocyanine (AlSPc) were coupled to polyclonal sheep anti-mouse-Ig antibody and bound to cells coated with specific mouse monoclonal antibody. When illuminated with red light, the AlSPc in the liposomes was activated to produce singlet oxygen and the antibody and liposome targeted cells were destroyed. DW-BCL cells (an Epstein Barr virus immortalised B-cell line) were targeted with an anti-B-cell antibody (8A) and killed specifically, both alone and in the presence of bone marrow mononuclear cells (BM-cells), without phototoxic effects on the untargeted bone marrow CFU-GM progenitor cells. The presence of an excess of non-target cells did not interfere with antibody and liposome binding, or light access to target cells. Similar results were obtained with T-lymphocytes as target cells using anti-CD3 antibody. Specific targeting to the B-cells was demonstrated in the cell mixtures by use of fluorescent microscopy combined with a sensitive technique to detect low levels of AlSPc fluorescence, a cooled charge couple device (CCD) camera. This was also able to show low levels of non-specific background binding of AlSPc to BM-cells and a small population of cells that took up AlSPc in the absence of antibody. The latter were shown to be monocytes by flow cytometry.

In the field of photodynamic therapy, there is a constant search for more efficient and specific photosensitisers. The phthalocyanines are a group of compounds which has excited interest as potentially useful photosensitisers and been much investigated over recent years (reviewed by Rosenthal, 1991). There is however, still scope for increasing the specificity of phthalocyanines for tumour tissue. Liposome incorporation is one useful possibility (Reddi *et al.*, 1987), and targeting with antibodies another. It has been previously shown that liposome encapsulated sulphonated aluminium phthalocyanine (AlSPc), is highly phototoxic when targeted to cells by antibody and activated by light (Morgan *et al.*, 1989). The phototoxicity of cells is probably due to a Type II photo-oxidation mechanism in which singlet oxygen is produced by energy transfer from light activated AlSPc to molecular oxygen (Sonoda *et al.*, 1987; Agarwal *et al.*, 1990). However, a contribution to cytotoxicity from a Type I mechanism cannot be ruled out (Kimel *et al.*, 1989; Ferraudi *et al.*, 1988). The singlet oxygen mechanism is most likely favoured over a Type I mechanism in this study because the AlSPc is in solution inside the liposome, not bound to a target substrate; and under the conditions of treatment there is no oxygen deprivation. Antibody specificity of the AlSPc liposomes was shown on single cell populations, with non specific antibodies and cell lines not expressing appropriate antigen acting as negative controls. The use of such liposomes as an *in vivo* therapy may not be effective because of their uptake in the reticuloendothelial system and limited access to target cells outside the vasculature. Therefore, it was postulated (Morgan *et al.*, 1989) that antibody targeted photosensitive liposomes might be a suitable agent for *ex vivo* use such as in the purging of residual tumour from bone marrow for autologous transplantation, and also for treatment for accessible diseases in anatomical compartments such as bladder carcinoma or tumours in the serous cavities. The present paper demonstrates the feasibility of targeting and destroying subpopulations of T-lymphocytes and B-cells (using an immortalised B-cell line as a model) in bone marrow with photosensitive liposomes without affecting normal bone marrow cells.

Correspondence: J. Morgan, Department of Urology, Henri Mondor Hospital, 94000, Creteil, France.
Received 31 January 1991; and in revised form 4 September 1991.

### Materials and methods

### *Cells*

DW-BCL is an *in vitro* Epstein Barr virus (EBV) immortalised polyclonal B-cell line (Azim, 1988). DW-BCL cells were seeded at $1 \times 10^5$ cells per ml and cultured in RPMI 1640 medium supplemented with 10% foetal calf serum.

Bone marrow was harvested from normal donors, or patients with non-Hodgkin's lymphoma in remission undergoing harvest for autologous bone marrow transplantation. Peripheral blood was collected from normal donors. The mononuclear cells of bone marrow (BM-cells), or peripheral blood (PBM) were obtained by layering the samples over Lymphoprep (Nycomed, Norway), centrifuging, and then collecting and washing the cells at the interface. In some experiments, the BM-cells were x-irradiated (3000 rad) to prevent cell division. T-lymphocytes were purified from PBM by incubating in 20% FCS, in a plastic petri-dish at 37°C for 4 h to allow monocytes to adhere. Non adherent cells were removed and were found to be greater than 95% positive for the CD3 antigen (a marker of T-lymphocytes) using UCHT1 antibody and flow cytometry.

### *Antibodies*

8A is an anti-B-cell mouse monoclonal antibody (subclass, IgG1), directed to an antigen expressed on B-cells from the pre-B stage of ontogeny through to plasma cells (Tazzari *et al.*, 1987). It is also expressed on other normal cells including some T-lymphocytes (personal observations) as well as DW-BCL cells. 8A was a gift from M. Gobbi and A. Bontadini, University of Bologna, Italy.

UCHT1 (subclass IgG1), is a mouse monoclonal antibody against the CD3 cluster antigen expressed on most peripheral blood T-lymphocytes.

UCHM1 (subclass IgG1), is a mouse monoclonal antibody against the CD14 antigen expressed on monocytes.

Sheep anti-mouse antibody (SαM), is a polyclonal antibody against mouse immunoglobulins produced in a sheep. This was affinity purified by absorption of the sheep serum against mouse immunoglobulins and also against human immunoglobulins to remove any cross reacting antibodies. UCHT1, UCHM1 and SαM were gifts from P. Beverley, ICRF, Human Tumour Immunology Group, UCL, London.

*Photosensitisers*

(i) Tetra-sulphonated AlSPc (tetra-AlSPc), prepared by the condensation method (Weber & Busch, 1965), was a gift from Russell Svenson, Royal Institution, London, UK.

(ii) TLC-AlSPc was a preparation made by the thin layer chromatographic separation of a mixture of tetra-, tri-, di- an mono-sulphonated AlSPc (a gift from Ciba-Geigy, Basel, Switzerland), as described in Morgan *et al.* (1989). The TLC preparation was enriched for the more hydrophilic tetra- and tri-sulphonated derivatives, with residual amounts of the more lipophilic di- and mono-sulphonated derivatives. HPLC traces run according to the method of Ambroz *et al.* (1991) (Figure 1), show the relative amounts of each component.

*Liposomes*

Liposomes were small unilamellar vesicles (SUV) with an average diameter of 50 nm, prepared by ultrasonication. They were coupled to SαM antibody with N-hydroxysuccinimidyl 3-(2-pyridyldithio)propionate (SPDP), (Sigma) in the presence of a buffer containing Hepes (10 mM) and NaCl (145 mM) by the method of Barbet *et al.* (1981). Lipids used were Lα-dipalmitoyl phosphatidylcholine (DPPC) and cholesterol (both from Sigma), and Lα-dipalmitoyl phosphatidylethanolamine-pyridyldithiopropionate in the ratio 66:33:1. The latter was derived from Lα-dipalmitoyl phosphatidylethanolamine (DPPE), (Calbiochem), using SPDP by the method of Barbet *et al.* (1981).

For each liposome preparation, $2 \times 10^{-5}$ moles of SαM containing a trace of $^{125}$I-labelled SαM were used for coupling to $4 \times 10^{-5}$ moles of total phospholipid. The radioactive trace was included to calculate the amount of antibody coupled to the liposomes. Tetra-AlSPc or TLC-AlSPc dissolved in Hepes buffer (pH 7.4) at 2 mM were encapsulated at the sonication stage. Control liposomes contained buffer only. The final concentration of SαM on the liposomes was $1.3 \pm 0.2 \times 10^{-6}$ M (mean ± s.e.m. of nine preparations) and the lipid concentration measured as DPPC (Stewart, 1980) was $3.4 \pm 0.18 \times 10^{-3}$ M (mean ± s.e.m. of seven preparations). The liposomes contained on average 47 molecules of tetra-AlSPc or 49 molecules of TLC-AlSPc. The liposomes were very stable to storage at 4°C in the dark; and over a period of 1 month leaked 5–6% of the contents. They were also stable under the cell treatment conditions, with no detectable AlSPc leakage after treatment with 7.2 J cm$^{-2}$ of light. The liposomes were not washed before use unless they had been stored for prolonged periods.

**Figure 1** HPLC traces of: **a**, unpurified Ciba Geigy preparation of sulphonated aluminium phthalocyanine and **b**, TLC purified preparation of **a** (TLC-AlSPc), showing the relative proportions of tetra-, tri-, di, and mono-sulphonated phthalocyanine (respectively 4S, 3S, 2S, 1S) present.

*Red light source*

The light source consisted of two 15 W Grolux fluorescent tubes (Thorn Lighting Ltd.) fitted with a red gelatin filter, model 182 (Lee filters Ltd.), as described by Chan *et al.* (1986), which gave peak emissions between 600 and 700 nm. The mean total light intensity (over all the transmitted wavelengths) was 2 mW cm$^{-2}$ as measured by a Coherent 212 Power meter. Cells were irradiated at 4°C to prevent thermal effects, the maximum increase of temperature being 4°C after 1 h illumination.

### Targeting and treatment procedures

*Cell targeting*

*Targeting and phototoxicity of DW-BCL cells alone* To determine the phototoxicity of AlSPc liposomes, DW-BCL cells were treated with combinations of antibody, liposomes and light under varying conditions.

For each experimental point, $1 \times 10^4$ cells harvested at the log phase of growth were added to triplicate flat bottomed wells in 96-well culture plates. They were incubated at 4°C with 50 μl antibody at 2 μg ml$^{-1}$ for 30 min, and then with 50 μl liposomes for 30 min, with three washes in cold serum free medium between each step.

Antibodies used were 8A (1/1000 dilution of ascites which contained approximately 2 μg ml$^{-1}$ of 8A) or UCHT1 (irrelevant control), followed by varying liposome dilutions. The liposomes were prepared with 2 mM tetra-AlSPc and different liposome dilutions were used to give a series of dose response curves. The lowest dilution of liposomes contained 0.3 nmoles of AlSPc per well (in a total volume of 50 μl) and doubling dilutions of this were made for the remainder, containing respectively 0.15, 0.08, and 0.04 nmoles. The highest AlSPc concentration corresponded to a 6 μmolar dose. Only the highest AlSPc concentration was used with the irrelevant antibody. Controls were incubated with antibody and buffer-only liposomes at similar lipid concentrations as those with AlSPc. Previous studies (unpublished work) have shown that the absolute number of liposomes in quantities similar to those used here, had no significant effect on cell growth; so buffer-only liposomes were only added in the same proportions as AlSPc-containing liposomes.

Before illumination DW-BCL cells were resuspended in a volume of 50 μl of RPMI medium without phenol red or FCS, and then exposed to red light for between 0 and 30 min to deliver total energies of up to 5.4 J cm$^{-2}$ as shown in Figure 2.

*Targeting and phototoxicity of T-lymphocytes* Isolated T-lymphocytes (2 million per treatment point) were incubated with UCHT1 at 2 μg ml$^{-1}$, and liposomes and exposed to red light in the same way as DW-BCL cells. Only one AlSPc concentration was used, at 0.3 nmoles, and one light dose, of 3.2 J cm$^{-2}$. Light was given in the absence of phenol red and FCS. The cells were then suspended at 2 million ml$^{-1}$ and

Figure 2 Effects of antibody bound tetra-AlSPc-liposomes on DW-BCL cells using an irrelevant antibody UCHT1 (Irrel Ab) and a specific antibody 8A at varying AlSPc-liposome dilutions, after illumination with red light. Each point is the mean ± s.e.m. of three experiments with triplicates at each point. —□— Irrel Ab; —Δ— 0.3 nmoles; —□— 0.15 nmoles; —Δ— 0.08 nmoles; —■— 0.04 nmoles.

Figure 3 Specific phototoxicity of 8A antibody bound AlSPc-liposomes to DW-BCL cells mixed with x-irradiated bone marrow mononuclear cells (BM-cells). 'Post-mixed' indicates cells mixed after targeting but before illumination and 'pre-mixed' indicates cells mixed before targeting. Also shown are the phototoxic effects of non-specifically bound liposomes made with the different preparations of photosensitisers: TLC-AlSPc and tetra-AlSPc (see text), and the effects of light alone on DW-BCL cells. Each point is the mean ± s.e.m. of three experiments with triplicates at each point. —□— Post-mixed; —▲— Pre-mixed; —□— TLC-AlSPc; —Δ— Tetra-AlSPc; —■— Light only.

incubated at 37°C until viability was measured by a Trypan blue exclusion test. Aliquots of the treated cell suspension were removed at different time periods after treatment, mixed with an equal volume of 0.2% Trypan Blue solution, and the number of viable cells, which excluded the dye were counted in a haemocytometer.

*Targeting and phototoxicity of DW-BCL cells or T-lymphocytes in a mixed population* Theoretically, the presence of an excess of non-target cells could limit the cytotoxic effect of liposomes on the target population by reducing either:

(i) Binding of antibody to target cells; or

(ii) The penetration of light to target cells.

This was tested for by the addition of excess non-target BM-cells. These tests for phototoxicity of mixed populations could then be compared with those for phototoxicity of single populations of DW-BCL cells (above). For these experiments, DW-BCL cells were therefore mixed with a 10-fold excess of BM-cells before targeting with antibody and liposomes (pre-mixed), and compared with cells mixed after targeting but before illumination (post-mixed).

For treatment of cell mixtures, x-irradiated BM-cells were added either before or after the incubation of DW-BCL with antibody and liposomes, to give a total of $1 \times 10^5$ cells per well (i.e. 10% of the total were DW-BCL). For experiments in which CFU-GM colonies (colony forming units of macrophages and granulocytes) were to be grown to determine survival of progenitor cells, non-x-irradiated BM-cells were used in similar proportions (see below).

The quantities of antibodies and liposomes used and the treatment procedure were similar to those for DW-cell alone, but only one concentration of tetra-AlSPc liposomes was used,, 0.3 nmoles. In addition, DW-BCL cells in BM-cells were treated with similar concentrations of TLC-AlSPc liposomes. Cells were resuspended in 50 µl of RPMI in the absence of phenol red or FCS and exposed to varying light doses of up to 3.6 J cm$^{-2}$ as shown in Figure 3.

T-lymphocytes already present in BM-cells were also targeted using UCHT1 and liposomes, but in this case, only CFU-GM colony recovery was assessed and no attempt was made to determine lymphocyte survival.

*Phototoxicity of CFU-GM progenitor cells* When the effects of the AlSPc-liposomes on the 'non-targeted' progenitor cells were to be examined the BM-cells were not x-irradiated, and the surviving cells were grown up as CFU-GM colonies. For these experiments the cells at $7.5 \times 10^5$ per well plus 10% added DW-BCL cells were incubated with antibody and/or liposomes as shown in Figure 4, and using similar quantities to previously, then exposed to a total of 0 or 3.6 J cm$^{-2}$ of light. Again, 2 mM tetra-AlSPc-liposomes were used at 0.3 nmoles of AlSPc per well, for all samples.

*Cell growth assays and viability*

The DW-BCL cell line is immortalised, but not transformed, and does not form colonies in soft agar. A tritiated thymidine incorporation assay was therefore used to assess the phototoxicity of this cell line. Surviving DW-BCL cells were grown after treatment by incubating in a humidified atmosphere with 5% $CO_2$ at 37°C for 72 h to allow time for the death of non-viable cells and the regrowth of cells capable of reproducing. They were pulsed with $^3H$ thymidine (Amersham) at 0.5 µCi well for the last 6 h, then harvested onto filters with an Automash cell harvester (Dynatech). The dried filters were transferred to vials, scintillation fluid was added, and the radioactivity in the samples was counted. In the experiments for examining the effects of photosensitive liposomes on DW-BCL cells in mixtures the BM-cells were x-irradiated and radioactive counts from controls containing only x-irradiated BM-cells were subtracted from those containing both DW-BCL cells and BM-cells.

**Figure 4** CFU-GM recovery from bone marrow mononuclear cells (BM-cells), alone or mixed with DW-BCL cells and treated with different mixtures as indicated and either exposed or not exposed to red light. Columns are means ± s.e.m. of four experiments using four different bone marrows with triplicate dishes for each treatment point. ■, UCHT1 only; ▩, 8A only; ▒, AlSPc-liposomes; ▨, UCHT1 + AlSPc-liposomes; □, 8A + AlSPc-liposomes.

For myeloid colony growth, a modification of the method of Pike and Robinson (1970) was used. DW-BCL/BM-cell mixtures or pure BM-cells in the case of T-lymphocyte targeted samples, were added after treatment to soft agar growth medium in 30 mm petri dishes to give a final number of $2 \times 10^5$ BM-cell per dish in 1 ml of medium. The agar medium consisted of Iscove's minimum Dulbecco medium (double strength), foetal calf serum and 0.9% agar dissolved in double distilled water, in the ratio 3:2.5:3, and contained 10% 5,637 bladder carcinoma cell line conditioned medium (Fraser *et al.*, 1988) to supply growth factors for colony growth. The dishes were incubated in a humidified atmosphere in 4% $CO_2$ for 14 days before the CFU-GM colonies were counted. A colony was scored when it contained more than 50 cells.

### Detection of specific targeting to cells

### *Detection by CCD camera*

To show that liposomes only bound to the target populations of cells, a sensitive technique for detecting small amounts of fluorescence from light activated AlSPc was used. This was a cooled charge-coupled device (CCD) camera, as described by Barr *et al.* (1988), and was used here with only slight modifications. Briefly, an inverted microscope with epifluorescence and phase contrast attachments was coupled with a cooled CCD camera (model 1, resolution 600 × 400 pixels, Wright Instruments). AlSPc bound to cells was excited with light from a 1 mW helium-neon laser tuned to 632.8 nm. The laser beam was focused through the objective and through a glass slide placed upside down on the stage onto the sample on the underside of the slide. AlSPc fluorescence between 665 and 700 nm was detected by the CCD camera using suitable band pass filters and processed by computer to produce a digital image. The computer also controlled the camera operation. Photographs of the fluorescent and phase contrast images of the same field of the slides were recorded from the screen.

Samples for CCD camera examination were prepared as follows: mixtures of bone marrow and B-cells incubated with antibody and liposomes were made in the same way as for the phototoxicity studies above, and fixed in 1% formaldehyde solution. Cytospin preparations were then made and the slides stored at 4°C in the dark until examination.

### *Flow cytometry*

To assess non-specific uptake of liposomes by DW-BCL/BM-cell mixtures the fluorescence of stained cells was examined by flow cytometry on a Coulter EPICS flow cytometer set up to detect red fluorescence from AlSPc and blue fluorescence from a second layer conjugate bound to monocyte-specific monoclonal antibody.

Samples of $2 \times 10^5$ cells were incubated for 30 min with 50 µl of liposomes containing 2 mM tetra-AlSPc, but with no SαM attached (to prevent non specific binding of antibodies in a subsequent incubation with a mouse monoclonal antibody). To determine whether cells which took up liposomes non-specifically in the first incubation were also monocytes, the cells were incubated with UCHM1 (cell hydridoma culture medium previously titrated to give optimum binding for monocytes in PBM), followed by the fluorescent second layer, Cascade Blue goat anti-mouse immunoglobulin (Molecular Probes, Eugene Oregon). Cells were washed after each step. Other samples were incubated either with liposomes only, with UCHM1 and Cascade Blue second layer only, or with Cascade Blue second layer only, for use as controls and setting gates on background red and blue fluorescence. Cells were finally fixed in medium containing 1% formaldehyde solution and their fluorescence measured.

Fluorescence was excited with uv light at 350 nm and detected using a 408 nm interference filter to block laser light, a 550 nm dichroate filter and a 630 nm longpass filter, thus allowing detecting of blue fluorescence due to Cascade Blue and red fluorescence due to AlSPc. Ten thousand cells were assessed on each run, on a linear scale.

### Results

Figure 2 shows the phototoxic effect of AlSPc liposomes on the growth of DW-BCL cell as a percentage of controls as measured by $^3H$ thymidine uptake. In the presence of light and a targeting antibody, $^3H$ thymidine uptake by DW-BCL cells is reduced to less than 1% of controls at the highest doses of tetra-AlSPc and light, whereas in the absence of targeting antibody, growth was not significantly affected (student's *t*-test) at any light dose.

$LD_{50}$ light doses for the different quantities of AlSPc added to cells were 0.73, 1.17, 1.26 and 2.33 J cm$^{-2}$ for 0.3, 0.15, 0.08 and 0.04 nmoles of AlSPc respectively. No $LD_{50}$ was obtained for non-specific toxicity because it did not reach such low levels, even at the longest light exposures used. Typical dose-response curves were obtained for dilutions of liposomes, showing decreasing growth inhibition for increasing liposome dilutions. This effect could be overcome completely by increasing the light exposure time for 0.15 nmoles of AlSPc, but not for the lower doses at the light exposures used here.

In the presence of x-irradiated BM-cells no significant difference (student's *t*-test) was observed between the phototoxic killing of DW-BCL cells regardless of whether the BM-cells were added before or after targeting of the B-cells with antibody and liposomes, Figure 3. This indicates that the presence of a 10 fold excess of non-target cells does not interfere with binding of antibody or light access to the target cells. In both cases a greater than two log kill was achieved with an $LD_{50}$ of 0.7 J cm$^{-2}$. There was no difference between tetra-AlSPc and TLC-AlSPc (data of latter not shown). Some non-specific toxicity of DW-BCL cells was observed when UCHT1 was used as an irrelevant antibody for both photosensitiser preparations at higher light doses. For tetra-AlSPc-liposomes the non-specific phototoxicity was only significant at light doses greater than $1.2\,J\,cm^{-2}$ ($0.005 < P \leqslant 0.01$), but not large enough to give an $LD_{50}$ value. However, when the TLC-AlSPc-liposomes were used, there was a much greater non-specific toxicity of DW-BCL cells at light doses of 1.2 J $cm^{-2}$ ($P \leqslant 0.0005$) and above with an $LD_{50}$ of $1.24\,J\,cm^{-2}$. Non specific toxicity of TLC-AlSPc liposomes were also significantly greater than that of the tetra-AlSPc at 2.4 J $cm^{-2}$ ($0.025 < P \leqslant 0.05$) and $3.6\,J\,cm^{-2}$ ($0.005 < P \leqslant 0.005$).

Table I shows the phototoxicity of T-lymphocytes over a period of 18 h after targeting and light exposure. Onset of photokilling is very rapid, with 30% of cells non-viable after 1 h, and very few alive after 18 h. Viability was not counted over longer periods of time because the untreated control cells also deteriorated with time.

Figure 4 shows the colony growth of bone marrow samples expressed as a percentage of untreated controls, after treatment with two different antibodies (8A and UCHT1) and AlSPc-liposomes in the presence or absence of light. Bone marrow treated with 8A contained 10% added target DW-BCL cells, whereas the normal CD3 positive component of bone marrow was the target for UCHT1. None of these treatments inhibited CFU-GM growth although they were effective against target cells (Figure 3, and Table I). There were no significant differences between any of the treatments, and no evidence of dark toxicity by AlSPc-liposomes. The non specific toxicity by AlSPc-liposomes observed for the DW-BCL cells (above) was not reflected in the recovery of bone marrow colonies.

Figure 5a shows the digital pattern of fluorescence produced by exciting AlSPc bound to B-cells cells in bone marrow by 8A antibody. There is a background population of faintly stained cells and some brightly fluorescing target cells. The total number of fluorescent cells was greater than the 10% of added DW-BCL because of the presence of some B-lineage cells and some T-lymphocytes (which bind 8A) among the BM-cells, and non-specific binding and uptake. Figure 5b shows AlSPc bound to cells non-specifically in the absence of antibody. The faintly stained background is present with the occasional bright cell (arrowed). The latter are probably due to uptake of liposomes by cells of monocytoid lineage in the BM-cells or the presence of peripheral blood macrophages taken up during aspiration of the bone marrow sample. It was not within the scope of this study to determine the exact populations of cells in BM-cells that bound liposomes *via* 8A antibody, but a brief study of the cells taking up AlSPc in the absence of antibody was done by flow cytometry.

Of the four DW-BCL/BM-cell mixtures examined, the results gave a range of 4–7% positive for UCHM1, and 6–12% positive for AlSPc above background, which was set at a level to give 1% positive cells for negative controls. Cells which were positive for both UCHM1 and AlSPc, corresponded to those of UCHM1 only, in the range 4–7% positive. Thus the majority of cells exhibiting non-specific uptake of AlSPc expressed a monocytic phenotype.

**Table I** Percentage viability of T-lymphocytes (s.e.m. < 10%)

| | *Time – hours after treatment* | | | | |
|---|---|---|---|---|---|
| *Treatment* | *1* | *2* | *3* | *6* | *18* |
| UCHT1 + AlSPc-liposomes-SαM | 70 | 59 | 37 | 12 | 4 |
| UCHT1 + AlSPc-lipsomes | 98 | 97 | 97 | 94 | 92 |
| Untreated | 99 | 99 | 98 | 98 | 97 |

The table shows the effects of antibody-bound tetra-AlSPc liposomes on the viability of isolated peripheral blood T-lymphocytes after red light illumination. Liposomes containing 2 mM tetra-AlSPc were conjugated to SαM and bound to T-lymphocytes by UCHT1 antibody. Control tetra-AlSPc liposomes were without SαM.

The viability of T-lymphocytes, measured by Trypan Blue exclusion, is expressed as a percentage of the starting number of cells for each point. Each figure is the mean of three experiments with duplicate counts at each time point. Standard errors of the mean were less than 10%.

**Figure 5** The photographs show the digital images of AlSPc fluorescence produced by laser excitation of tetra-AlSPc in liposomes, bound specifically to B-cells with anti-B-cell antibody 8A **a**; and bound or taken up non-specifically in the absence of targeting antibody **b**. In both cases, the cells were a mixture of 10% DW-BCL cells in BM-cells.

### Discussion

Many patients with haematological malignancies (or solid tumours) who are either unsuitable for allogeneic bone marrow transplantation or do not have HLA-matched donors, could benefit from the escalation of therapy which is made possible by an autologous bone marrow transplant (ABMT). Autologous bone marrow is harvested prior to intensive therapy following which high dose chemo- or radiotherapy is given to destroy the tumour. The doses given are also marrow ablative. The stored marrow is then reinfused, and after engraftment haematological function is restored. However, autologous bone marrow may be contaminated with clonogenic tumour cells, even though they may be undetectable morphologically, thereby leading to relapse after ABMT.

To try to prevent this, various methods have been used in an attempt to purge bone marrow of residual tumour cells. These include various pharmacological, mechanical and immunological techniques each of which has its proponents.

An animal model (Sharkis *et al.*, 1980) showed that leukaemic relapse could be prevented by treating bone marrow with the cytotoxic agent 4-HC (4-hydroperoxycyclophosphamide). Another study showed a possible benefit to some patients with acute non-lymphoblastic and lymphoblastic leukaemia purged with Asta Z 7557, but there was a wide range of sensitivity to the drug and individual doses were determined by pre-testing with bone marrow aspirates (Gorin *et al.*, 1986). A further study showed some evidence of improved disease-free survival in patients with acute myeloblastic leukaemia who received AMBT with grafts purged by 4-HC (Rowley *et al.*, 1989). More recently, Gorin *et al.* (1990), made a multi-centre retrospective analysis of ABMT in Europe to investigate the benefits of mafosfamide purged *vs* non-purged marrow in acute myeloid leukaemia and found a higher leukaemia-free survival and lower probability of relapse with purging. There are, therefore, some indications that purging, even using such non-specific drugs may have a beneficial effect, although no prospective randomised clinical trial has demonstrated such a benefit.

The problem with pharmacological agents is their adverse effect on haemopoietic stem cells as well as tumour stem cells, which may retard both the onset and extent of haematological reconstitution, an undesirable situation for immunologically compromised patients who have undergone ablative regimes as part of their therapy. Ideally, more selective agents would be of benefit, with a greater differential between tumour kill and stem cell toxicity, resulting in better therapeutic index.

Photosensitisers of various types have previously been shown to have differential toxicity between haemopoietic and tumour progenitor cells, including merocyanine 540 (Sieber *et al.*, 1987) which is now being evaluated in phase I clinical trials, Photofrin II (Atzpodien *et al.*, 1987), and sulphonated aluminium phthalocyanine (Singer *et al.*, 1988). However, all of these photosensitisers showed considerably toxicity to haemopoietic stem cells.

The present study has shown no phototoxicity of CFU-GM progenitors, combined with high toxicity of targeted cells which suggests that a targeted method is more selective in action against tumour cells, with a better therapeutic index than treatment with free photosensitiser.

Sulphonated aluminium phthalocyanine was chosen for this investigation because it is available in polar forms which are hydrophilic and therefore suitable for encapsulating in liposomes. Tetra-AlSPc liposomes gave low levels of non-specific toxicity (Figure 3) compared to TLC-AlSPc liposomes which also contained small amounts of the less polar, lower sulphonated derivatives (as shown in Figure 1). It is likely that there is the same amount of non-specific adherance to cells for both tetra-AlSPc and TLC-AlSPc liposomes. However, the tetra-AlSPc, being more highly sulphonated, and thus more polar than some of the components in TLC-AlSPc, is relatively less able to cross the lipid bilayer of the lipsome and pass into the cell membrane, thus producing less toxicity. Greater non specific toxicity was noted in the DW-BCL/BM-cell mixtures than when the DW-BCL cells alone were targeted. This may be due to the presence of AlSPc bound non-specifically to the large excess of BM-cells. Similar $LD_{50}$ results were obtained for both specific DW-BCL cell toxicity in marrow (0.70 J $cm^{-2}$) and DW-BCL cells alone (0.73 J $cm^{-2}$) using 0.3 nmoles of AlSPc. Previous results obtained for the same dose of AlSPc on DW-BCL when illuminated in the presence of growth medium (RPMI with phenol red and 10% FCS) gave an $LD_{50}$ of 0.9 J $cm^{-2}$, an increase of 23% (results not shown). A further series of eight experiments comparing phototoxicity of DW-BCL treated with 0.3 nmoles of AlSPc and 3.6 J $cm^{-2}$ of light in the presence and absence of growth medium, showed a mean increase of cell survival of 27.5 ± 6.9% in the former case. It is obviously of great importance that interfering substances are minimised during the light activation stage. Both phenol red and FCS interfere slightly with light penetration by absorbing light. At the main absorption peak of AlSPc (675 nm) which is also one of the main emission peaks of the light source, absorbance due to phenol red and FCS amounts to 1.1%, with the phenol red contribution being 0.4%. This is not sufficient to account for the difference in cellular phototoxicity obtained in the presence and absence of these agents. Human plasma (Kanofsky, 1990), and FCS (Parker & Stanbro, 1984), have been shown to quench singlet oxygen. The presence of FCS in the medium would therefore be expected to inhibit the action of the singlet oxygen produced, and protect against its toxicity. It is unlikely that the effects of serum were due to any direct interaction with AlSPc, since it is protected from the external medium by the lipid bilayer.

It is interesting that no toxicity occurred to CFU-GM progenitors, despite the presence of low levels of non-specifically bound AlSPc as shown in Figure 5. This may be due to a lower susceptibility of progenitors to oxidative damage. Seven day CFU-GM colonies which represent a later, more committed progenitor were not counted because the clusters and colonies were too numerous, which indicates that it is not just the earlier progenitors that were protected. This contrasts with another study also using 8A antibody to target cells (Dinota *et al.*, 1990), but using free radicals produced by the enzyme activity of xanthine oxidase on a substrate in the medium, in which 7 days, but not 14 days CFU-GM were affected, though their results were otherwise similar to ours.

The antibody targeted liposome system as described here for destroying specifically targeted cells in bone marrow bone appears to have many advantages as a bone marrow purging agent. AlSPc is not toxic until it is activated by red light, and then the onset of photodamage is very rapid as indicated by the decrease in viability of T lymphocytes in Table I. Large quantities of photosensitiser may be encapsulated in the liposomes and an additional amplification occurs during irradiation because each photosensitiser molecule is 'reusable' and can be activated several times over, producing large amounts of the toxic species single oxygen for relatively small quantities of AlSPc. Furthermore the toxic reactions is not dependent on cells being in an active phase of cycling, as demonstrated by the antibody targeted phototoxicity of unstimulated T-lymphocytes. This means that clonogenic cells in Go can be targeted and destroyed. The T-lymphocytes in this study were obtained from 'non-responders'. These are donors whose T-lymphocytes are not activated via the CD3 pathway in response to the binding of anti-CD3 antibodies, in this case of subclass IgG1 (Smith *et al.*, 1986), thus ensuring that the lymphocytes were quiescent during treatment.

The use of a polyclonal antibody on the liposomes means that several mouse monoclonal antibodies can be used together as a cocktail with the same AlSPc preparation. Also, because the AlSPc containing liposomes need not be internalised to be toxic (Morgan *et al.*, 1989), since the singlet oxygen produced is able to diffuse towards the cell, many cell surface antigens that would not normally be internalised can be used to extend the number of potential binding sites on the target cells. A combination of antibodies against both endocytosing and non-endocytosing antigens might be the best option to use, to attack the cell from both the inside and the outside.

We would like to thank Bloomsbury Health Authority for the grant that supported this work. Grateful thanks are also due to Arnold Pizzey who set up and ran the EPICS for the flow cytometry.

### References

AGARWAL, R., ATHAR, M., BICKERS, D.R. & MUKHTAR, H. (1990). Evidence for the involvement of singlet oxygen in the photodestruction by chloroaluminium phthalocyanine tetrasulphonate. *Biochem. Biophys. Res. Comm.*, **173**, 34.

AMBROZ, M., BEEBY, A., MACROBERT, A.J., SIMPSON, M.S.C., SVENSON, R.K. & PHILLIPS, D. (1991). Preparative, analytical and fluorescence spectroscopic studies of sulphonated aluminium phthalocyanine photosensitisers. *J. Photochem. Photobiol. B: Biol.*, **9**, 87.

ATZPODIEN, J., GULATI, S.C., STRIFE, A. & CLARKSON, B.D. (1987). Photoradiation models for the clinical ex vivo treatment of autologous bone marrow grafts. *Blood*, **70**, 484.

AZIM, T. (1988). PhD Thesis, University of London.

BARBET, J., MACHY, P. & LESERMAN, L.D. (1981). Monoclonal antibody covalently coupled to liposomes: specific targeting to cells. *J. Supramol. Struct. Cell Bioch.*, **16**, 237.

BARR, H., TRALAU, C.J., MACROBERT, A.J., MORRISON, I., PHILLIPS, D. & BOWN, S.G. (1988). Fluorescence photometric techniques for determination of microscopic tissue distribution of phthalocyanine photosensitisers for photodynamic therapy. *Lasers Med. Sci.*, **3**, 81.

CHAN, W.S., SVENSON, R., PHILLIPS, D. & HART, I.R. (1986). Cell uptake, distribution and response to aluminium chloro sulphonated phthalocyanine, a potential antitumour photosensitiser. *Br. J. Cancer*, **53**, 255.

DINOTA, A., TAZZARI, P.L., ABBONDANZA, A., BATTELLI, M.G., GOBBI, M. & STIRPE, F. (1990). Bone marrow purging by a xanthine oxidase-antibody conjugate. *Bone Marrow Transplant*, **5**, 31.

FERRAUDI, G., ARGUELLO, G.A., ALI, H. & VAN LIER, J.E. (1988). Types I and II sensitised photooxidation of aminoacid by phthalocyanines: a flash photochemical study. *Photochem. Photobiol.*, **47**, 657.

FRASER, J.K., LIN, F. & BERRIDGE, M.V. (1988). Expression and modulation of specific high affinity binding sites for erythropoietin on the human erythroleukaemic cell line K562. *Blood*, **71**, 104.

GORIN, N.C., DOUAY, L., LAPORTE, J.P. & 15 others (1986). Autologous bone marrow transplantation using marrow incubated with Asta Z 7557 in adult acute leukaemia. *Blood*, **67**, 1367.

GORIN, N.C., AEGERTER, P., AUVERT, B. & 19 others (1990). Autologous transplantation for acute myelocytic leukaemia in first remission: a European survey of the role of marrow purging. *Blood*, **75**, 1606.

KANOFSKY, J.R. (1990). Quenching of singlet oxygen by human plasma. *Photochem. Photobiol.*, **51**, 299.

KIMEL, S., TROMBERG, B.J., ROBERTS, W.G. & BERNS, M.W. (1989). Singlet oxygen generation of porphyrins, chlorins and phthalocyanines. *Photochem. Photobiol.*, **50**, 175.

MORGAN, J., GRAY, A.G. & HUEHNS, E.R. (1989). Specific targeting and toxicity of sulphonated aluminium phthalocyanine photosensitised liposomes directed to cells by monoclonal antibody *in vitro*. *Br. J. Cancer*, **59**, 366.

PIKE, B.L. & ROBINSON, W.A. (1970). Human bone marrow colony growth in agar-gel. *J. Cell. Physiol.*, **76**, 77.

PARKER, J.G. & STANBRO, W.D. (1984). Dependence of photosensitised singlet oxygen production on porphyrin structure and solvent. In: *Porphyrin localisation and treatment of tumours*, Doiron, D.R. & Gomer, C.J. (eds), p. 259. Alan R. Liss Inc., New York.

REDDI, E., CASTRO, G.L., BIOLO, R. & JORI, G. (1987). Pharmacokinetic studies with zinc(II)-phthalocyanine in tumour-bearing mice. *Br. J. Cancer*, **56**, 597.

ROSENTHAL, I. (1991). Phthalocyanines as photodynamic sensitizers. *Photochem. Photobiol.*, **53**, 859.

ROWLEY, S.D., JONES, R.J., PIANTADOSI, S. & 8 others (1989). Efficacy of ex vivo purging for autologous bone marrow transplantation in the treatment of acute nonlymphoblastic leukaemia. *Blood*, **74**, 501.

SHARKIS, S.J., SANTOS, G.W. & COLVIN, M. (1980). Elimination of acute myelogenous leukaemic cells from marrow and tumour suspensions in the rat with 4-hydroperoxycyclosphophamide. *Blood*, **55**, 521.

SIEBER, F. (1987). Marrow purging by merocyanine 540-mediated photolysis. *Bone Marrow Transplant*, **2**, (Suppl 2) 29.

SINGER, C.R.J., LINCH, D.C., BOWN, S.G., HUEHNS, E.R. & GOLDSTONE, A.H. (1988). Differential phthalocyanine photosensitisation of acute myeloblastic leukaemia progenitor cells: a potential purging technique for autologous bone marrow transplantation. *Br. J. Haematol.*, **68**, 417.

SMITH, K., AUSTIN, J.M., HARIRI, G., BEVERLEY, P.C.L. & MORRIS, P.J. (1986). T-cell activation by anti-T3 antibodies: comparison of IgG 1 and IgG2b switch variants and direct evidence for accessory function of macrophage Fc receptors. *Eur. J. Immunol.*, **16**, 478.

SONODA, M., MURALI KRISHNA, C. & RIESZ, P. (1987). The role of singlet oxygen in the photohemolysis of red blood cells sensitised by phthalocyanine sulphonates. *Photochem. Photobiol.*, **46**, 625.

STEWART, J.C.M. (1980). Colorimetric determination of phospholipids with ammonium ferrothiocyanate. *Anal. Biochem.*, **104**, 10.

TAZZARI, P.L., GOBBI, M., DINOTA, A. & 7 others (1987). Normal and neoplastic plasma cell membrane phenotype. Studies with new monoclonal antibodies. *Clin. Exp. Immunol.*, **70**, 192.

WEBER, J.H. & BUSCH, D.H. (1965). Complexes derived from strong field ligands. XIX. Magnetic properties of transition metal derivatives of 4,4′,4″,4‴-tetrasulphophthalocyanine. *Inorg. Chem.*, **4**, 469.